# Surgical Treatment of Vascular Intramedullary Spinal Cord Lesions

**DOI:** 10.7759/cureus.3154

**Published:** 2018-08-16

**Authors:** George M Ghobrial, Jason Liounakos, Robert M Starke, Allan D Levi

**Affiliations:** 1 Neurosurgery, Novant Health, Miami, USA; 2 Neurosurgery, University of Miami, Miami, USA; 3 Neurosurgery, University of Miami Miller School of Medicine, Miami, USA

**Keywords:** intramedullary, spinal cord tumor, cavernoma, hemangioblastoma, spinal arteriovenous malformation

## Abstract

Background

Vascular lesions represent a rare subset of intramedullary spinal cord pathology and consist of cavernous malformations (CM), hemangioblastomas, and arteriovenous malformations (AVM). These lesions are each unique and the literature pertaining to their surgical management is largely limited to retrospective case series and case reports.

Objectives

To evaluate the surgical management of each of these lesions with special attention to postoperative functional status.

Methods

A single-institution case series of intramedullary vascular lesions treated with surgery was retrospectively evaluated. The primary variables of interest included preoperative and postoperative McCormick grades. Other variables of interest included frequency and indication for conventional spinal angiography, rates of preoperative embolization, postprocedural complications, operative time, intraoperative blood loss, and length of hospital stay.

Results

Thirty-six patients were identified over the 17-year study period, including 20 with hemangioblastomas, 13 with CMs, and three with AVMs. The median preoperative McCormick grades were 2, 2, and 3 for hemangioblastomas, CMs, and AVMs, respectively. The median postoperative McCormick grades were 2, 2, and 2 for hemangioblastomas, CMs, and AVMs, respectively at the most recent follow-up. Preoperative angiography was performed in all AVM cases and 29% of hemangioblastomas. Preoperative embolization was performed in 40% of hemangioblastoma cases undergoing preoperative angiography. Operative times were similar between the three lesion groups. In three cases of hemangioblastoma resection and one case of CM resection, McCormick grade improved by one point following surgery. At a mean follow-up of 30.9 months for hemangioblastomas, 7.95 months for CMs, and 24 months for AVMs, all patients were at least at their discharge baseline, with no new neurologic complaints.

Conclusion

Intramedullary vascular lesions are rare and represent a complex surgical patient population. Surgical resection with or without preoperative angiography and embolization appears to be safe and to halt neurologic decline.

## Introduction

Vascular lesions represent a rare and diverse subset of intramedullary spinal cord pathology consisting of cavernous malformations (CMs), hemangioblastomas, and arteriovenous malformations (AVMs). CMs comprise 5–12% of all spinal intramedullary lesions, making them the most common spinal vascular lesion [1]. Even so, they are not frequently encountered in practice with an overall incidence of 0.4–0.5% [2]. Hemangioblastomas are somewhat less common vascular neoplasms that account for 1.6–15% of intramedullary spinal cord lesions [3]. Spinal AVMs are the rarest subset of vascular lesions of the spinal cord, accounting for only 3–4% of all intramedullary lesions [4]. It was not until the advent of diagnostic spinal angiography techniques in 1977 did they become distinguishable from dural arteriovenous fistulas [5].

Given their relative rarity, the literature for the management of these lesions exists primarily in the form of retrospective clinical case series [6]. It is imperative to appreciate, separately, the unique angioarchitecture of each lesion, as well as their natural history, symptomatic presentation, diagnosis, indications for surgery, and surgical outcomes. Herein the authors present a clinical case series of the surgical management of intramedullary spinal cord cavernous malformations, hemangioblastomas, and intramedullary arteriovenous malformations over a 17-year period at a single institution.

## Materials and methods

Patients aged 18 and older were consecutively identified from a single institution, single surgeon (Allan D. Levi, M.D.) database that underwent surgical treatment for intramedullary hemangioblastomas, cavernous malformations, and arteriovenous malformations from January 1, 2000 until December 31, 2017. Preoperative examinations were evaluated and classified based on the clinical outcomes scale proposed by McCormick [7] for intramedullary tumor resection with grading as shown in Table 1. Institutional review board approval was obtained.

**Table 1:** McCormick Scale for Functional Classification of Intramedullary Spinal Cord Tumors.

Grade	Definition
1	Neurologically intact with normal ability to ambulate and minimal dysesthesia
2	Mild sensorimotor deficit, functionally independent
3	Moderate sensorimotor deficit, functionally independent with external aid
4	Severe sensorimotor deficit, functionally dependent
5	Paraplegic or quadriplegic

Diagnostic workup

All patients with a suspected lesion of the neuro-axis underwent preoperative magnetic resonance imaging (MRI) with thin-cut axial views of the suspected level. In the instance of MRI evidence of a hemangioblastoma or cavernous malformation, the remaining neuro-axis was subsequently evaluated by MRI to rule out lesional multiplicity. Diagnostic spinal angiography was performed in the case of a suspected hemangioblastoma to characterize the blood supply and potentially provide adjunctive embolization, especially with MRI evidence of hypervascularity. In the case of cryptic hemorrhage, spinal angiography was carried out in order to rule out a vascular malformation. Spinal angiography was not performed in the diagnosis of CM, as they are better characterized by T2-weighted imaging and are angiographically occult.

Surgical technique

For all intramedullary spinal cord tumors, patients were placed prone on a radiolucent Jackson table frame (Mizuho OSI, Union City, CA). Total intravenous anesthesia with propofol and opioid analgesia were utilized in order to facilitate motor and somatosensory evoked potentials, as well as electromyography. Antibiotics were administered within one hour prior to incision as per institution protocol. Preoperative localization of the lesion was performed with the use of intraoperative fluoroscopy. Upon localization of the appropriate levels, a subperiosteal dissection and laminectomy was performed. Prior to durotomy, confirmation of rostral and caudal exposure above and below the intramedullary lesion was confirmed with the use of intraoperative ultrasound. With the aid of an operating microscope, CMs and hemangioblastomas underwent extracapsular resection wherever possible, such as with an extracapsular hematoma occasionally seen with CMs. This strategy aims to minimize disruption of intervening spinal cord parenchyma, thereby sparing eloquent afferent and efferent tracts. In the absence of a lesion approaching the surface of the spinal cord, a posterior midline myelotomy was preferred for either posterior or deeper midline lesions with the use of the dorsal median sulcus and preservation of uninvolved pial vasculature. Hemangioblastomas can most often be approached via a dorsal midline approach since the majority are posteriorly located [8]. Ideally, for lateral lesions and more ventrally located lesions in symptomatic patients with CMs, a targeted myelotomy performed as described by Mitha et al. provided the most direct surgical corridor [9]. Often when a CM is ventral, the lesion is approached ventral to the dentate ligament, midway between the ventral and dorsal nerve roots.

## Results

Thirty-six patients from 2000 to 2017 were identified as undergoing surgical resection of an intramedullary vascular lesion. Their baseline characteristics are described in Table 2. Median preoperative McCormick grade was 2 for hemangioblastoma, 2 for CM, and 3 for intramedullary AVM. There were 20, 13, and three patients identified with hemangioblastomas, CMs, and intramedullary AVMs, respectively. Four patients with hemangioblastomas had a known diagnosis of von Hippel-Lindau (VHL) syndrome (20%), two of which had undergone previous posterior fossa craniotomy for intracranial hemangioblastoma resection. Symptomatic hemorrhage on presentation was present in one patient with a Type IV AVM (33%) and one patient with a hemangioblastoma (5%), while micro-hemorrhages were present on MRI in seven patients with CM (53.8%).

**Table 2:** Preoperative Patient Characteristics. SD: Standard deviation; IQR: Interquartile range.

Parameter	Hemangioblastoma	Cavernous Malformation	Arteriovenous Malformation
Number of Patients (n)	20	13	3
Age (Years; mean ± SD)	47.2 ± 16.3	50.5 ± 13.7	46.3 ± 22.0
Preoperative McCormick Grade (Median; IQR)	2; 2	2; 1	3; 1.5
American Society of Anesthesiologists (ASA) Score (Median; IQR)	2; 0.75	2; 1	3; 0.5

Preoperative imaging and spinal angiography

Diagnostic and surgical management characteristics are outlined in Table 3. All spinal intramedullary AVMs were preoperatively evaluated with spinal angiography. Three were identified and all were classified as Type IV. The presenting clinical findings included bilateral lower extremity numbness, variable signs and symptoms of mild thoracic myelopathy, and acute paraplegia due to a spontaneous hemorrhage. Profound T2-weighted hyperintensity of the spinal cord with dilated dorsally located intradural veins was noted in all three cases. Preoperative embolization was not performed in any case. Preoperative spinal embolization was performed in two of five (40%) spinal angiograms performed on patients with hemangioblastomas. One of the two vertebral feeders was selectively catheterized and coil embolized in one patient resulting in a reduction in tumor vascularity. In another patient, a right costocervical branch supplying the tumor was coil embolized. No permanent decline in neurologic exam was noted in either patient. Embolization could not be achieved in one patient due to a common origin with the anterior spinal artery, and in the other two no safely catheterizable feeder vessels were identified.

**Table 3:** Diagnostic Features and Surgical Management Characteristics. SD: Standard deviation

Parameter	Hemangioblastoma	Cavernous Malformation	Arteriovenous Malformation
Patients Undergoing Preoperative Angiography (Percent of Total n)	5 (29%)	0 (0%)	3 (100%)
Patients Undergoing Preoperative Embolization	2	0	1
Number of Laminectomy Levels (mean ± SD)	2.75 ± 1.02	2.69 ± 0.480	2.67 ± 0.577
Cervical Location of Lesion (Percent of Total n)	17 (85%)	9 (69%)	0
Thoracic Location of Lesion (Percent of Total n)	1 (5%)	4 (31%)	3 (100%)
Lumbar Location of Lesion (Percent of Total n)	2 (10%)	0 (0%)	0
Operative Time (Minutes; mean ± SD)	403.8 ± 126.9	310.1 ± 155.9	386.3 ± 135.8
Blood Loss (mL; mean ± SD)	285 ± 217.4	Insufficient Data	250 ± 173.2

Surgical characteristics

The mean number of laminectomies performed ranged from 2.67 to 2.75. Eighty-five percent of hemangioblastomas were localized to the cervical spine, whereas 69% and 31% of CM were localized to the cervical and thoracic spine, respectively. All AVM cases were localized to the thoracic spine. The mean intraoperative blood loss was 285 mL for hemangioblastomas and 250 mL for intramedullary AVMs. Intraoperative blood loss for CM was not documented adequately to be reported here. Mean operative duration ranged from 310 minutes for CM to 386.3 minutes and 403.8 minutes for AVM and hemangioblastomas, respectively (Table 3).

Postoperative course

The mean hospital length of stay was longest for intramedullary AVMs (9 ± 3.61 days), followed by hemangioblastomas (7.47 ± 4.61 days), and CMs (5.25 ± 2.49 days), albeit with a high degree of variability. The median postoperative McCormick Scale by discharge was 2, 2, and 3 for hemangioblastomas, CMs, and intramedullary AVMs, respectively (Table 4). In two of 13 (15.4%) patients with CM, neurologic status declined by 1 point on the McCormick Scale. All other patients remained unchanged or demonstrated improvement at discharge. At the latest follow-up, aside from two patients with hemangioblastoma who declined one point on the McCormick Scale, all patients remained at least at their discharge baseline. There were no instances of postoperative hemorrhage. Residual gadolinium enhancement was seen in 15% of hemangioblastoma and CM cases. Residual T2 hyperintensity was seen in the spinal cord in 52.3%, 38%, and 67% of hemangioblastoma, CM and AVM cases, respectively.

**Table 4:** Surgical/Clinical Results and Postoperative Course. SD: Standard deviation; IQR: Interquartile range.

Parameter	Hemangioblastoma	Cavernous Malformation	Arteriovenous Malformation
Postoperative McCormick Grade at Discharge (Median; IQR)	2; 2	2; 1	3; 1.5
McCormick Grade at Latest Follow-up (Median; IQR)	2; 1.75	2; 1.5	2; 1
Hospital Length of Stay (Days; mean ± SD)	7.47 ± 4.61	5.25 ± 2.49	9 ± 3.61
Follow-up (Months; mean ± SD)	30.9 ± 49.5	7.95 ± 7.24	24 ± 1.41
Cases with Residual Gadolinium Enhancement on Follow-up (Percent of Total n)	3 (15%)	2 (15%)	Data not available
Cases with T2 Hyperintensity on Follow-up (Percent of Total n)	11 (52.3%)	5 (38%)	2 (67%)

## Discussion

Intramedullary vascular lesions are an interesting and rare subset of spinal pathology. Previously, surgical case series have reported excellent surgical outcomes with the resection of cavernous malformations [10], hemangioblastomas [11, 12], and intramedullary AVMs [13, 14]. The purpose of this study is to add our experience over 17 years to the growing body of knowledge related to the surgical management of these lesions, with particular attention paid to functional outcomes.

Cavernous malformation

Cavernous malformations represent the most common intramedullary vascular spinal cord lesions. Their propensity for hemorrhage is related to identified genetic mutations of the CCM1, CCM2 and CCM3 genes, resulting in irregularly thin-walled, sinusoidal arrangements of blood vessels [10]. Four clinical presentations are evident with CMs as described by Ogilvy et al. [15]: discrete episodic neurologic decline with varying degrees of recovery, slow, progressive neurologic decline, acute onset of symptomatology with rapid decline, or acute symptom onset with a slow, progressive course. The acuity and rate of clinical progression is thought to relate to the degree and manner of hemorrhage within the lesion. Micro-hemorrhages may contribute to progressive lesion growth and while less often, larger hemorrhages may result in acute and rapid neurologic decline [16]. In our series micro-hemorrhages were a common finding on preoperative MRI. Transient symptoms, characterized by a relapsing and remitting course attributed to micro-hemorrhage within the CM, make the decision on when to move forward with surgical resection difficult. All patients with CMs were symptomatic in our series, with surgical resection resulting in radiographic cure in the vast majority of cases. Median McCormick grade remained stable, as compared to preoperative grade, at the most recent follow-up (mean: 7.95 months). Overall, 63% of patients presented with motor deficits. While evidence in the literature is scarce regarding the natural history of untreated intramedullary CMs, a low risk of clinically significant hemorrhage amounting to less than 1% per year has been quoted as an argument for observation [17]. Yet another challenge lies in how to manage a symptomatic patient with multiple lesions throughout the neuro-axis [9].

In a 2010 meta-analysis of the retrospective surgical series that comprises the majority intramedullary CM management literature [1], 352 CMs were identified in the thoracic spine most frequently (57%), followed by the cervical spine (38%). Interestingly, in our series, the vast majority of lesions were located in the cervical spine (69%). The specific location of CMs can vary, making surgical resection high risk for postoperative motor weakness due to interruption of the descending corticospinal tract fibers. On MRI a heterogeneous core surrounded by a T2 hypointense periphery (an artifact of hemosiderin deposition) is characteristic [18]. These lesions are angiographically occult, and as such no patient with a CM underwent a diagnostic angiogram in our series.

Hemangioblastoma

As with CMs, there is a limited consensus regarding the surgical management of asymptomatic hemangioblastomas [19]. Many surgeons find exception with one autosomal dominant familial cancer syndrome, VHL, which has been associated with nearly 30% of all hemangioblastomas and accounts for a more rapidly progressive tumor. Due to variable expressivity seen in VHL, the rate of growth frequently is unpredictable [6]. Regardless of tumor growth, VHL-associated hemangioblastomas may be resected to provide a surgical cure [8], while maintaining a low morbidity and excellent prognosis. In our series, a total of 20 patients underwent surgery, with one patient undergoing a second hemangioblastoma resection for a separate lesion. The clinical presentation varies based on the location of the tumor nidus within the spinal cord, as well as the presence and rate of cyst growth [19].

Preoperative spinal angiogram is an effective method to delineate the anatomy of these lesions, but many surgeons argue that the benefit is limited due to the relatively smaller tumor size encountered in the spinal cord compared to intracranial lesions of the same variety [8]. In the largest series in the literature, with a total of nine patients, Eskridge et al. [12] report the successful use of preoperative embolization to lower intraoperative blood loss. In one case, however, neurologic decline was noted due to tumor swelling and acute hydrocephalus from complete necrosis of the large nidus. In all cases, an avascular nidus was noted following onyx embolization, even in the same patients where prior surgical resections were aborted due to excessive blood loss. Only two hemangioblastomas were safely embolized in our study. For large and complex lesions, this may decrease perioperative blood loss, but care should be taken, as embolization may result in lesional swelling and associated complications of embolization [20].

As an alternative, Lonser and Oldfield proposed several key maneuvers to aid in hemangioblastoma resection [21]. Initially, the margins of the tumor-pial interface are delineated by bipolar coagulation. Capsular identification is facilitated by incising the pia just lateral to the junction to avoid entry into the tumor, minimizing blood loss. Circumferential dissection is performed, and individual vessels are coagulated and cut with microscissors in order to disconnect the capsule from the spinal cord [21].

Arteriovenous malformation

Only three patients with spinal AVM undergoing surgery were identified, limiting the depth of observations that can be made. All three were type IV AVMs. It is the authors’ opinion that all patients should undergo spinal angiography to define high-risk features, including associated aneurysms, venous varices, stenosis, and/or thrombosis. One patient with thoracic myelopathy underwent a spinal angiogram, demonstrating a T10 fistulous point with a dorsal tangle of perimedullary veins. No embolization was performed due to close association with a dorsal spinal artery. In one patient, coil embolization of feeders was performed successfully, but a complete angiographic cure was unable to be achieved due to proximity to the anterior spinal artery.

No clear guidelines exist in the literature pertaining to the preoperative or postoperative angiographic evaluation of these lesions. The use of adjunctive preoperative embolization is primarily described in retrospective clinical case reports. In a series of 17 patients treated by Corkill et al. using Onyx embolysate, a 37.5% obliteration rate was reported [22]. Embolization may be curative in more simple lesions, but overall obliteration rates are low and can be associated with significant complications. For extremely complex lesions that cannot be safely removed with microsurgery, palliative embolization may slow the fistula and temper venous hypertension, resulting in symptomatic improvement [23-25]. With improvements in endovascular technology, embolization may play a growing role in the treatment of select lesions [26].

Limitations

Due to relatively small number of patients included in this observational study, statistical comparison between the three groups of vascular spinal cord lesions could not be carried out. Future guidance and understanding of the clinical presentation, patient population, diagnostic workup, interventional and surgical management and outcomes, and postoperative course will likely be obtained from meta-analysis of the literature and large registries.

## Conclusions

Intramedullary vascular lesions are rare and represent a complex surgical patient population. Surgical resection with or without preoperative angiography and embolization appears to be safe, and likely halts neurologic worsening. With regard to preoperative diagnostic angiography or embolization, the benefit of these adjuncts is not well defined, with the exception of intramedullary AVMs, where many consider angiography the initial diagnostic modality of choice. Prospective, cohort-matched studies are required to evaluate surgical or angiographic intervention in this patient population.
